# The Impact of a Multi-Strain Probiotic Supplementation on Puppies Manifesting Diarrhoeic Symptoms During the Initial Seven Days of Life

**DOI:** 10.3390/ani15121700

**Published:** 2025-06-09

**Authors:** Piotr Andrzej Socha, Barbara Maria Socha

**Affiliations:** 1 Department of Animal Reproduction with Clinic, Faculty of Veterinary Medicine, University of Warmia and Mazury in Olsztyn, Oczapowskiego Str. 14, 10-719 Olsztyn, Poland; 2 Centrum Zdrowia Zwierząt Veterinary Clinic, Bogusławskiego Str. 2E/1, 11-300 Biskupiec, Poland; barbara.m.socha@gmail.com

**Keywords:** multi-strain probiotic, diarrhoea, puppies, dog, Labrador Retriever

## Abstract

Acute diarrhoea is a prevalent condition in puppies, frequently accompanied by a reduction in daily weight gain and an elevated risk of mortality. The utilisation of probiotics has been demonstrated to facilitate the restoration of intestinal microflora homeostasis. To date, limited research has investigated the effect of probiotics on reducing diarrhoea in very young puppies fed exclusively on mother’s milk. The aim of the present study was to evaluate the impact of administering a multi-strain probiotic on reducing diarrhoeic symptoms in puppies during their first seven days of life. A total of 77 Labrador Retriever puppies, aged between one and seven days, were examined. The study found that 55% of the puppies presented with clinical symptoms of diarrhoea. The puppies exhibiting diarrhoea were administered a multi-strain probiotic comprising the *Lactobacillus plantarum* AMT4 and AMT14 strains and the *Bifidobacterium animalis* AMT30 strain for a period of five days in monotherapy. The results demonstrated that within 24 h of the first dose, a reduction in the incidence of diarrhoeic symptoms was observed in 47.62% of the dogs. Following a 72 h period, 83.34% of the puppies exhibited a resolution of their symptoms. The animals that were part of the study demonstrated expected weight gain and were in good condition. The preliminary study confirmed the high effectiveness of the multi-strain probiotic in halting diarrhoea in puppies up to seven days of age. The results obtained from this study are highly promising and provide new perspectives on the treatment of diarrhoea in puppies fed exclusively on their mother’s milk.

## Simple Summary

Acute diarrhoea is one of the most common reasons for veterinary consultations in dogs. In puppies, it is often associated with a decrease in daily weight gain. It is particularly dangerous in very young animals due to the increased risk of mortality. Diarrhoea is often non-specific, with an unknown or multifactorial cause, and its occurrence indicates a disturbance in the homeostasis of the intestinal microflora, i.e., dysbiosis. The use of probiotics has been shown to facilitate the restoration of intestinal microflora homeostasis. Probiotics are defined as live microorganisms that, when consumed in adequate amounts, may exert a beneficial effect on the health of the host. To date, limited research has investigated the effect of probiotics on reducing diarrhoea in very young puppies fed exclusively on mother’s milk. The aim of this study was to evaluate the effect of administering a multi-strain probiotic on reducing the symptoms of diarrhoea in puppies during the first seven days of life. The preliminary study confirmed the high efficacy of a probiotic containing lactic acid bacteria strains of *Lactobacillus* and *Bifidobacterium* in stopping diarrhoea in puppies up to seven days of age. The results are very promising and offer new perspectives in the treatment of diarrhoea in puppies fed exclusively on mother’s milk.

## 1. Introduction

Acute diarrhoea is among the most common causes of veterinary consultations in dogs [1]. Puppies under one year of age are particularly vulnerable to gastrointestinal infections, with diarrhoea representing a common and potentially serious condition. A decline in daily weight gain and an elevated risk of mortality have been documented in cases where this condition has been observed [2].

Diarrhoea is a multifactorial condition, with a wide variety of bacterial (e.g., *Clostridium perfringens*, β-haemolytic *Eschericha coli*), viral (e.g., Canine parvovirus type 2—CPV2, Canine coronavirus—CCV), and parasitic (e.g., *Giardia* spp.) agents, which are described as enteropathogens in puppies [1,2,3]. Furthermore, the combination of nutritional and lifestyle factors with environmental stressors has been identified as a contributing factor to gastroenteritis in puppies [1]. In dogs, intestinal chronic or acute inflammation alters the composition of the intestinal microbiota [1]. Although acute diarrhoea is usually self-limiting, treatment is commonly administered in an attempt to lessen the severity or duration of the condition. Probiotics, antibiotics, and dietary modifications, either alone or in combination, are frequently employed for this purpose [4]. The commonplace usage of antibiotics for treating a self-limiting condition raises concerns regarding appropriate antimicrobial stewardship because this practice could promote bacterial resistance [4,5]. Consequently, a multifaceted approach is imperative to prevent diarrhoea, which must include the administration of probiotics, particularly during the weaning period [2].

Probiotics are defined as live microorganisms that, when consumed in adequate amounts, may exert a beneficial effect on the health of the host. The mechanisms by which probiotics exert their effects include the displacement of pathogenic microorganisms, the improvement of epithelial barrier function, the facilitation of the assimilation of vitamins and nutrients, and the modulation of the enteric and innate immune responses [1,3,6,7,8,9,10]. In light of the widely acknowledged ramifications of antibiotic utilisation, there has been a resurgence of interest in probiotics, which have been demonstrated to engender physiological health benefits for the host [1]. To date, probiotics, particularly those containing *Lactobacillus* and *Bifidobacterium* strains that have been approved for use in dogs [11], have been demonstrated to confer a range of health benefits to the host, including the ability to restore equilibrium to the gut microbiota [1]. However, the impact of bacterial strains on the treatment of diarrhoea during the initial days of life in canine puppies fed exclusively on their mother’s milk has yet to be investigated in the extant literature.

In light of these findings, the objective of this study was to evaluate the impact of administering a multi-strain probiotic comprising the *Lactobacillus plantarum* AMT4 and AMT14 strains and the *Bifidobacterium animalis* AMT30 strain on reducing diarrhoeic symptoms in puppies during their first seven days of life.

## 2. Materials and Methods

### 2.1. Animal and Material Collection

**Figure 1:**
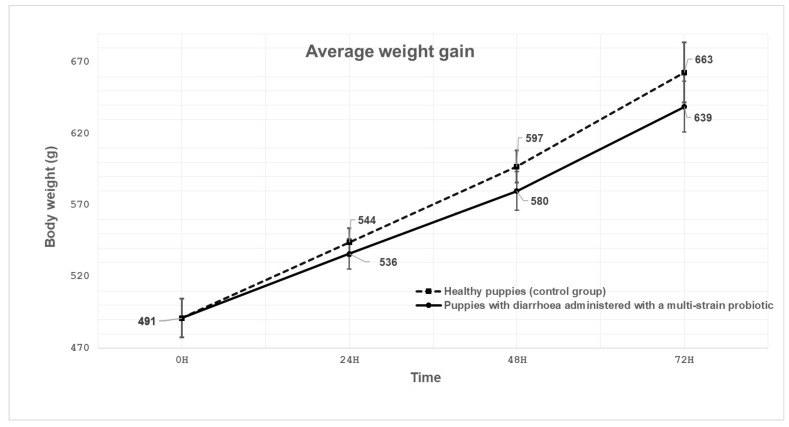
The mean daily weight gain of healthy puppies and puppies exhibiting symptoms of diarrhoea that were administered a multi-strain probiotic.

A total of 11 privately owned Labrador Retriever litters, for which the results of faecal examinations for parasites and viruses in both puppies and their mothers were confirmed to be negative, were selected for inclusion in the study between June and September 2023. A total of 77 puppies, aged between 1 and 7 days, underwent examination. The puppies were delivered naturally. The mean birth weight of the puppies was recorded as 420 g, and the changes in weight gain in both healthy puppies (control group) and those with diarrhoea treated with probiotic are presented in a growth curve (Figure 1). The experiment examined the rate of recovery from diarrhoea in puppies, as indicated by changes in body weight gain, compared with healthy puppies of the same age. While a placebo-controlled design is typically preferred, ethical constraints regarding neonatal puppies and the risk of exacerbating diarrhoea symptoms in untreated animals precluded the use of a placebo group. The puppies were fed exclusively on their mother’s milk. The bitches were vaccinated, dewormed, and fed in accordance with standard veterinary practice. Puppies exhibiting signs of diarrhoea were administered a multi-strain probiotic (PetBIOM, Owlie S.A., Vet No. αPL2814117p, Stawiguda, Poland) containing the *Lactobacillus plantarum* AMT4 and AMT14 strains, as well as the *Bifidobacterium animalis* AMT30 strain. The probiotic preparation was administered after the puppies were weighed, with the dosage amounting to an average of 750 mg to 1000 mg, depending on the body weight of the examined puppies. The powder preparation was dissolved in a minimal volume of water and administered twice daily, with an interval of 12 h between doses, for a period of five days as monotherapy. A multi-strain probiotic was administered with the consent of the animal owners as the study included client-owned animals. Informed consent was obtained from the owners for the inclusion of their dogs in this study and for the collection of faecal samples and the administration of multi-strain probiotics, either verbally or in writing, prior to the start of the study. The procedures were performed for clinical indications as part of routine veterinary practice. This was conducted in accordance with the accepted ethical standards and generally accepted principles of good medical practice, in adherence to a high standard (best practice) of veterinary care. According to Polish legislation, activities carried out within the scope of veterinary practice are not subject to the approval of the Ethical Committee (The Polish Act on the Protection of Animals Used for Scientific or Educational Purposes, adopted on 15 January 2015 (Journal of Laws 2015, item 266)).

### 2.2. Faecal Samples Examination

Prior to the selection of the group, all faecal samples, obtained from both puppies and their mothers, were collected immediately following defecation and divided into three parts. The initial portion of the faecal samples (faecal swabs) was subjected to analysis through the utilisation of a rapid diagnostic test, specifically the Rapid CPV/CCV Antigen Test, with the objective of promptly excluding the potential presence of viral diseases.

The second part of the samples was placed in sterile plastic stool containers and transported on ice to the laboratories of the Department of Parasitology and Invasive Diseases of the Faculty of Veterinary Medicine, University of Warmia and Mazury in Olsztyn. There, an initial macroscopic examination was conducted to monitor colour, sample texture, the presence of blood, the presence of adult/larval helminths, and the presence of proglottids of tapeworms. Subsequently, all samples were subjected to examination through flotation and sedimentation techniques. The presence of parasite developmental forms was detected through the use of Giemsa staining. Moreover, the faecal samples were subjected to examination through the implementation of a one-step rapid diagnostic assay for the detection of *Giardia* sp., *Leishmania* sp., and *Cryptosporidium* sp. A flotation procedure was conducted by combining a 1 g faecal sample with 5 mL of Darling solution. Subsequently, the sample was poured through a tea strainer into a beaker or faecal cup, after which it was subjected to centrifugation at 2500 rpm for a period of five minutes. The centrifuged samples were then examined under a light microscope at 10× and 40× magnification. In the sedimentation technique, a small volume of the sediment was placed on a slide with a long coverslip, and the sample was examined under a light microscope at 10× and 40× magnification.

The third part of the faecal samples (faecal swabs) was submitted for microbiological examination to the Department of Microbiology and Clinical Immunology, Faculty of Veterinary Medicine, University of Warmia and Mazury in Olsztyn. The faecal swabs were pre-incubated in non-selective tryptic soy broth (Oxoid, Basingstoke, UK) at 37 °C for 24 h under aerobic conditions. The samples were then inoculated onto Columbia agar supplemented with 5% defibrinated sheep blood (Oxoid, Basingstoke, UK), MacConkey (Oxoid, Basingstoke, UK), Edwards (Oxoid, Basingstoke, UK) and Chapman (Oxoid, Basingstoke, UK) agar. Bacteria were cultured under aerobic conditions at 37 °C for 48 h. The grown isolates were subjected to microbiological analysis. The morphology of bacterial colonies, Gram staining, selected biochemical tests (catalase, coagulase and oxidase tests; API 20E and API 20NE tests) (bioMérieux, Lyon, France), CAMP reaction and selected latex tests (PathoDxtra Strep grouping kit, Staphytect Plus) (Oxoid, Basingstoke, UK) were conducted. Culturing for anaerobic bacteria was performed in CHROMagar™ *C. perfringens* medium (ChromAgar, Saint-Denis, France). Anaerobic cultures were incubated at 37 °C in an anaerobic chamber with the Gaspak™ system (Oxoid, Wokingham, UK). Subsequently, grown colonies were identified by Gram staining and the API 20A test.

### 2.3. Multi-Strain Probiotic

The probiotic preparation used in this study has only recently been employed in veterinary medicine. As stated in the manufacturer’s leaflet, PetBIOM (Owlie S.A., Vet No. αPL2814117p, Stawiguda, Poland) is a supplementary feed mixture formulated for administration to dogs and cats. The product has been developed for use in veterinary gastroenterology, with indications including diarrhoea, belching, munching, vomiting, abdominal pain, bloating in the abdomen, excessive gases, intestinal dysbiosis, antibiotic therapy, inflammatory bowel diseases, and so forth. The probiotic preparation PetBIOM contains 200 billion (2 × 10^11^ cfu/kg) beneficial bacteria strains, i.e., the *Lactobacillus plantarum* AMT4 strain, the *Lactobacillus plantarum* AMT14 strain and the *Bifidobacterium animalis* AMT30 strain. The distinctiveness of these strains has been formally acknowledged by the patent offices of Poland (No. P.414701, P.419333), the European Union (No. EP3168292) and the USA (No. 20180303884). The strains incorporated within the PetBIOM formulation have been designated as GRAS (Generally Recognised As Safe), thereby attesting to their suitability for utilisation in animals.

### 2.4. Statistical Analysis

The statistical analysis was conducted using SigmaPlot Software Version 12.0 (Systat Software Inc., San Jose, CA, USA). Gaussian distribution was tested using the D’Agostino & Pearson normality test. The Shapiro–Wilk test was performed to test the normality of the data. Presented data are the mean ± SEM from 35 animals (control group) or 45 animals (experimental group including puppies with diarrhoea supplemented with a multi-strain probiotic). The mean daily weight gain of healthy puppies and puppies with diarrhoea were compared using Mann–Whitney U test at three time points (24, 48 and 72 h) followed by Dunn’s multiple comparisons test. The results were considered significantly different at a *p*-value < 0.05.

The mean body weights of both groups are presented in Figure 1. The graphical presentation of the daily weight gains was created using Microsoft Excel software (Microsoft Office 365, Redmond, WA 98052-7329, USA).

Table 1 presents data demonstrating the effect of a multi-strain probiotic (PetBIOM) on the reduction in diarrhoea in puppies at 24, 48, and 72 h. The data are presented in two formats: as absolute values for individual litters and as percentages of the total number of puppies exhibiting diarrhoea at three time points.

## 3. Results

### 3.1. The Impact of a Multi-Strain Probiotic Comprising the Lactobacillus plantarum AMT4 and Lactobacillus plantarum AMT14 Strains and the Bifidobacterium animalis AMT30 Strain on Reducing Diarrhoeic Symptoms in Puppies Aged Between 1 and 7 Days

Table 1 demonstrates the efficacy of a multi-strain probiotic in reducing diarrhoea in several-day-old puppies at 24, 48 and 72 h post-administration.

Of the 77 Labrador Retriever puppies from 11 litters, 54.54% (42/77) exhibited clinical signs of diarrhoea, while the remaining 45.45% (35/77) were healthy and served as the control group. A reduction in the incidence of diarrhoeic symptoms was observed in 47.62% of the dogs (20/42) within 24 h of the initial dose of the multi-strain probiotic. After 48 h, a 69.05% reduction in symptoms was observed (29/42), with a further 83.34% reduction evident after 72 h. Notably, in five of the eleven litters, all puppies exhibited complete resolution of diarrhoea within 72 h.

### 3.2. Evaluation of Daily Weight Gain of Puppies Supplemented with a Multi-Strain Probiotic

The mean daily weight gain of healthy puppies (control group) and those exhibiting signs of diarrhoea that were administered a multi-strain probiotic is demonstrated in Figure 1. The mean body weights of the puppies were compared at the three time points (24, 48 and 72 h) during the course of the experiment. The statistical analysis revealed no significant differences at three time points between the healthy puppy group (control group) and the group of puppies exhibiting signs of diarrhoea and administered a multi-strain probiotic (*p* > 0.05).

## 4. Discussion

The findings of this study indicate that probiotic treatment has the potential to promote self-healing and likely reduce the duration of diarrhoea in puppy dogs diagnosed with acute nonspecific diarrhoea. A recent placebo-controlled study on adult dogs by Herstad et al. [12] concluded that probiotics may reduce the duration of clinical signs in dogs administered a combination probiotic for the treatment of acute diarrhoea from multiple causes. However, it should be noted that probiotic effects are likely to be condition- and strain-specific [10].

The benefits of probiotics in animal feed have been extensively documented; however, research on the use of probiotics in the treatment of diarrhoea in puppies fed exclusively on mother’s milk is limited. The majority of extant publications pertain to studies in adult dogs and the treatment of diarrhoea with metronidazole, amoxicillin or supportive probiotics [1,4,5,13]. In the study conducted by Shmalberg et al. [13], it was determined that the time to resolution of diarrhoeal signs was found to be comparable for different substances. Adult dogs with a body mass ranging from 4 to 45 kg exhibited an acceptable faecal consistency after an average of 3.5 ± 2.2 days following probiotic administration, 4.6 ± 2.4 days with oral metronidazole, and 4.8 ± 2.9 days with placebo. What is important that statistically significant differences were not identified between the treatment groups (*p* = 0.17). A similar subject, but in puppies aged between one and four months, has been researched by Molina et al. [1]. The study revealed a swift recovery in puppies diagnosed with acute diarrhoea, when administered intravenous fluids, an antiparasitic agent, amoxicillin, and enrofloxacin, in conjunction with multi-strain probiotic therapy [1].

Our study is the first to report the benefits of administering a multi-strain probiotic to very young puppies (aged 1 to 7 days) suffering from diarrhoea. While a placebo-controlled design is typically preferred, ethical constraints regarding neonatal puppies and the risk of exacerbating diarrhoea symptoms in untreated animals precluded the use of a placebo group. Therefore, healthy puppies from the same examined litters were the control group in this study. Throughout the study, all supplemented puppies were in good condition, nursing well, and gaining weight and had no clinical complications. The resolution of diarrhoeal symptoms in the puppies was comparable to the results obtained by other authors in adult dogs undergoing antibiotic therapy or probiotic-assisted self-medication [1,4,13]. It is noteworthy that no fatalities in puppies were documented in this study, which is likely attributable to the beneficial effects of probiotic supplementation.

A number of factors have been demonstrated to exert an influence on the gastrointestinal tract and faecal microbiome of canines. These factors include age, sex, breed, diet and environmental conditions [1,3,10,14,15]. The microbiome of the puppy is initiated during the foetal developmental phase, with a significant increase in the number of beneficial *Lactobacillus* strains occurring within the first 48 h of life. Disturbances occurring during the early stages of development have been demonstrated to result in a significant decrease in the population of bacteria of this species. This, in turn, may subsequently lead to the development of dysbiosis in the initial days of life [2]. Such disturbances may result in the development of gastrointestinal disorders, including diarrhoea. Diarrhoea is a multifactorial condition prevalent in puppies, with a wide variety of bacterial, viral and parasitic agents having been implicated in its aetiology [1,2,3]. Furthermore, the utilisation of antibiotic therapy is not advised in the treatment of such conditions in dogs that are very young in age. In the study conducted by Werner et al. [5], it was demonstrated that treatment with amoxicillin–clavulanic acid offers no clinical benefit to canines afflicted with acute diarrhoea. However, it was found that this treatment predisposes the development of amoxicillin-resistant *E. coli*, which have been observed to persist for a duration of up to three weeks following the conclusion of treatment. These findings support international recommendations that dogs with diarrhoea should not be treated with antimicrobials unless there are signs of sepsis. In our opinion, this is particularly important in the treatment of puppies that are immature and susceptible to dysbiosis.

In the present study, an examination of the faeces of the puppies and their mothers for the presence of parasites (*Giardia* sp., *Leishmania* sp., *Cryptosporidium* sp., tapeworms and roundworms) and viruses (CPV and CCV) yielded negative results. Furthermore, a bacterial culture of the faecal samples obtained from the puppies manifesting diarrhoeic symptoms indicated the presence of predominantly *Clostridium perfringens* (41 in out of 42 puppies) and *Escherichia coli* (42 in out of 42 puppies). Moreover, faecal examination from among 42 puppies with diarrhoea indicated the presence of *Klebsiella pneumoniae* (in seven puppies), *Staphylococcus pseudointermedius* (in four puppies), *Pseudomonas aeruginosa* (in three puppies) and *Staphylococcus canis* (in one puppy). Observing the development of the canine neonate microbiome, in the study conducted by Guard et al. [16], *Clostridiaceae* family members are prominent at day 2 of puppies age and then decreased over time. *Clostridium perfringens* is a normal commensal of the gastrointestinal tract of healthy individuals; however, its overgrowth has been demonstrated to play a significant role in canines suffering from diarrhoea [5,17,18]. The present study suggests that the aetiology of diarrhoea in puppies is the result of dysbiosis and disturbances in the composition of the developing intestinal microflora of puppies. The most prevalent bacterial components detected in faeces from puppies with diarrhoea symptoms were *C. perfringens* and *E. coli*. Furthermore, *E. coli* is a component of the normal intestinal flora in canines and can also be isolated from the vagina in healthy female animals. However, it is also frequently diagnosed as the causative agent in swabs taken from diseased puppies or post-mortem samples [19]. The aetiology of *E. coli* in newborn puppies may be attributable to exposure to the faeces of the dam and the puppy during the process of delivery, as has been observed in human cases [20]. Further studies are required to evaluate the role of various bacterial strains in puppies in the first days of life, either in healthy subjects or in those with diarrhoea.

Probiotics can be categorised according to a variety of criteria, including their type and origin. The predominant category of probiotics are lactic acid bacteria, encompassing *Lactobacillus* spp., *Bifidobacterium* spp., and *Streptococcus* spp. [21]. In dogs, variable effects have been observed for the strains from the following bacterial genera: *Lactobacillus*, *Enterococcus*, *Bacillus, Streptococcus*, *Bifidobacterium*, *Pediococcus*, and *Weissella* [9,22,23]. In the study conducted by Pascher et al. [24], a decline in the numbers of *Clostridium perfringens* and *Escherichia* spp. was reported in six German Shorthair Pointers following the administration of *Lactobacillus acidophilus*. In the present study, it was hypothesised that the inhibition of diarrhoea in puppies with probiotic supplementation was attributable to the inhibition of bacterial overgrowth in the gastrointestinal tract of puppies. Nevertheless, it is imperative that further microbiological studies are conducted in order to provide unequivocal confirmation of this hypothesis.

In turn, in the study by Xu et al. [25], it was demonstrated that the oral administration of the *Lactobacillus casei* Zhang, *Lactobacillus plantarum* P-8 and *Bifidobacterium animalis* subsp. *lactis* V9 strains exerted a beneficial effect on canines, with the observed effects varying according to the age of the animals. The study revealed enhancements in food intake, body weight gain, immunity and intestinal microbiota. The results of the present study also indicate a beneficial effect on supplementation with probiotics. There were no differences in daily weight gain between the groups of healthy puppies and puppies with signs of diarrhoea treated with a multi-strain probiotic.

Furthermore, a recent study conducted on lambs demonstrated that the oral administration of a multi-strain probiotic comprising *Lactobacillus plantarum* AMT4 and AMT14 strains and *Bifidobacterium animalis* AMT30 strain exerts a beneficial impact on the phagocytic activity and oxidative metabolism of peripheral blood granulocytes and monocytes after stimulation with *E. coli* bacteria [21]. It suggest that the tested probiotic formulation may have a positive effect on the immune status of lambs [21]. Whilst the results pertain to a different species, they are consistent with the observations made in the present study regarding the utility of the selected probiotic strains employed in PetBIOM including the *Lactobacillus plantarum* AMT4 strain, the *Lactobacillus plantarum* AMT14 strain, and the *Bifidobacterium animalis* AMT30 strain. Namely, in our study, a significant percentage of puppies exhibited the complete resolution of diarrhoeal symptoms by the third day of therapy, indicating the beneficial effects of the *Lactobacillus plantarum* AMT4 and AMT14 strains and the *Bifidobacterium animalis* AMT30 strain in reducing the symptoms of acute nonspecific diarrhoea. Moreover, the findings of our previous report demonstrated the beneficial effect of the same multi-strain probiotic (PetBIOM, Owlie S.A., Vet No. αPL2814117p, Poland) on the composition of vaginal flora in reproductive bitches [26]. Following probiotic administration, alterations in the composition of the vaginal flora were observed. The frequency of Gram-negative rods other than *E. coli* was found to be significantly reduced. The elimination of potential pathogens such as *Clostridium perfringens*, *Canicola haemoglobinophilus* and *Pseudomonas aeruginosa* was achieved. Consequently, the results of studies on multi-strain probiotics containing lactic acid bacteria strains, such as the *Lactobacillus plantarum* AMT4 and AMT14 strains and the *Bifidobacterium animalis* AMT30 strain, in ameliorating the symptoms of acute non-specific diarrhoea in puppies are encouraging. However, further clinical studies utilising advanced laboratory techniques are required to substantiate these findings and to ascertain the efficacy of the administered bacterial strains. 

Despite the intriguing results obtained from this study, it is important to acknowledge some limitations of the study design. Firstly, it is important to note that no microbiome testing was performed either before or after treatment. This could provide a potential explanation for the observed effect of supplementation with a probiotic containing lactic acid bacteria strains, such as *Lactobacillus* and *Bifidobacterium*, on potentially pathogenic bacterial genera, including *Clostridium*. Secondly, the study did not include full faecal microbiome sequencing, which would have been capable of detecting changes in the composition of the gut microbiome in puppies supplemented with a multi-strain probiotic. Thirdly, the implementation of polymerase chain reaction (PCR) testing for parasites in faecal swab samples could be performed on account of the fact that it is both highly sensitive and requires a smaller quantity of material for molecular biology testing. Furthermore, the proprietors administered the treatments in the comfort of the patients’ own homes. Despite undergoing training to administer the powder in accordance with the prescribed schedule and to report instances where administration of the probiotic was not feasible, it cannot be entirely discounted that in certain cases, the treatment was not administered. In addition, the clinical activity of the dogs was assessed by the owners themselves. It is acknowledged that whilst some of the variables included in the results can be objectively assessed (e.g., stool consistency using a stool scoring system), some of the variables (e.g., activity, condition) are relatively subjective. The clinical impression is also contingent upon the duration of observation of the puppies, a factor that varies between owners. Moreover, it is important to note that the full generalisation of the results is not possible, at least in part, due to the fact that all of the canines included in the study were from a single geographical region. The authors are cognisant of the primary limitation of this study, namely the absence of a placebo or blinded group. In future studies on this topic, the addition of a control group of animals would be necessary to obtain adequate power between probiotic and placebo treatment. This approach has the potential to engender a stratification of the recovery process by emphasising the underlying causes of recovery. However, it appears improbable that the outcomes would undergo substantial modification in the presence of a placebo group. The limitations described herein are consistent with the multifactorial nature of diarrhoea and its treatment, as well as the inherent difficulties in assessing puppies, especially in the first days of life. This study, when considered in conjunction with other extant data, may contribute to the development of guidelines for the use of probiotic acid bacteria in the treatment of acute diarrhoea in puppies.

## 5. Conclusions

A multi-strain probiotic was found to be highly efficacious in the prevention of acute non-specific diarrhoea in puppies up to seven days of age. A notable improvement was observed in a considerable number of cases within 24 h of probiotic supplementation. The majority of puppies exhibited complete resolution of the symptoms by the third day of the treatment period. At the final examination, conducted five days after the initial assessment, all puppies were found to be in good clinical condition. Although a study with a placebo control group is usually preferred, ethical restrictions on neonatal puppies and the risk of exacerbation of diarrhoeal symptoms in untreated animals prevented the use of a placebo group. Nonetheless, the results obtained in this study are very promising and offer new insights into the management of diarrhoea in puppies fed exclusively on mother’s milk and substantiate the efficacy of the multi-strain probiotic formulation used. These encouraging outcomes warrant further investigation to elucidate the precise impact of the *Lactobacillus plantarum* AMT4 and AMT14 strains and the *Bifidobacterium animalis* AMT30 strain on the treatment of gastrointestinal disorders in puppies within the first days of life.

## Figures and Tables

**Table 1:** The effectiveness of a multi-strain probiotic in the treatment of diarrhoea in the puppies at 24, 48 and 72 (h) after administration.

Litter ID	Number of Puppies	Number of Healthy Puppies (Control Group)	Number of Puppies with Diarrhoea	Number of Puppies with Diarrhoea 24 h After a Multi-Strain Probiotic Administration	Number of Puppies with Diarrhoea 48 h After a Multi-Strain Probiotic Administration	Number of Puppies with Diarrhoea 72 h After a Multi-Strain Probiotic Administration
1	11	5	6	3	2	0
2	9	4	5	5	3	1
3	9	5	4	2	0	0
4	8	3	5	2	1	1
5	6	2	4	2	1	1
6	6	3	3	1	0	0
7	3	1	2	1	0	0
8	8	5	3	1	0	0
9	6	2	4	2	1	1
10	6	3	3	1	0	0
11	5	2	3	2	1	0
	77	35	42 (100%)	22 (52.38%)	9 (21.42%)	4 (9.52%)

## Data Availability

None of the data were deposited in an official repository.
